# Meningiomas of the Anterior Clinoid Process: Is It Wise to Drill Out the Optic Canal?

**DOI:** 10.7759/cureus.321

**Published:** 2015-09-10

**Authors:** Michael Sughrue, Ari Kane, Martin J Rutkowski, Mitchel S Berger, Michael W. McDermott

**Affiliations:** 1 Neurosurgery, University of Oklahoma; 2 Department of Radiology, Duke University Medical Center; 3 Department of Neurological Surgery, University of California, San Francisco

**Keywords:** meningioma, clinoid meningioma, optic canal, meningioma surgery

## Abstract

*Introduction*: Meningiomas of the anterior clinoid process are uncommon tumors, acknowledged by most experienced surgeons to be among the most challenging meningiomas to completely remove. In this article, we summarize our institutional experience removing these uncommon and challenging skull base meningiomas.

*Methods*: We analyzed the clinical outcomes of patients undergoing surgical removal of anterior at our institution over an 18-year period. We characterized the radiographic appearance of these tumors and related tumor features to symptoms and ability to obtain a gross total resection. We also analyzed visual outcomes in these patients, focusing on visual outcomes with and without optic canal unroofing.

*Results*: We identified 29 patients with anterior clinoid meningiomas who underwent surgical resection at our institution between 1991 and 2007. The median length of follow-up was 7.5 years (range: 2.0 to 18.6 years). Similar to others, we found gross total resection was seldom safely achievable in these patients. Despite this, only 1/20 of patients undergoing subtotal resection without immediate postoperative radiosurgery experienced tumor progression. The optic canal was unroofed in 18/29 patients in this series, while in 11/29 patients it was not. Notably, all five patients experiencing visual improvement underwent optic canal unroofing, while three of four patients experiencing visual worsening did not.

*Conclusions*:  These data provide some evidence suggesting that unroofing the optic canal in anterior clinoid meningiomas might improve visual outcomes in these patients.

## Introduction

**Figure 1:**
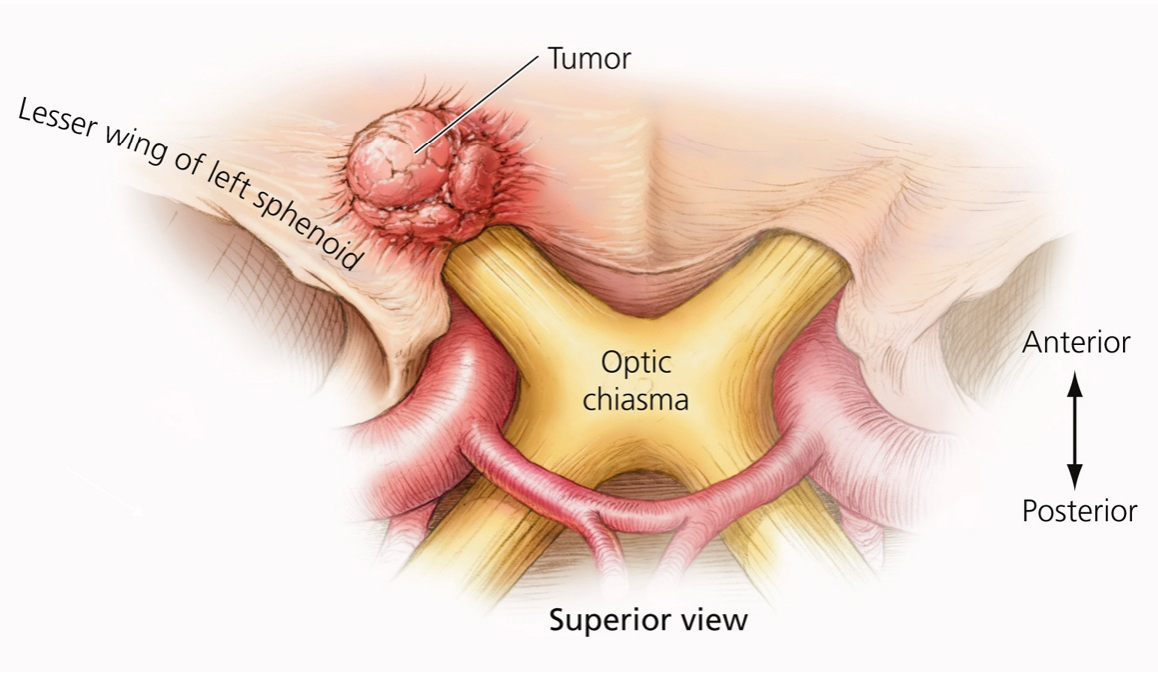
Axial illustration of small clinoid meningioma Clinoid meningiomas arise from the medial aspect of the sphenoid wing and grow superiorly from the clinoid region.

**Figure 2:**
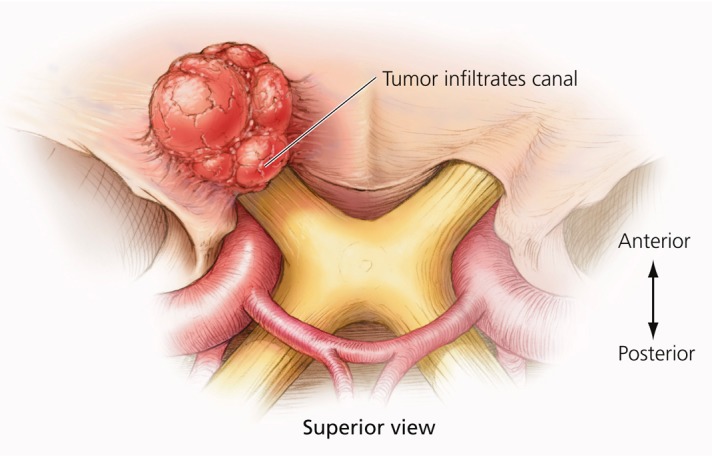
Growing tumor with optic canal invasion With tumor growth, extension into the optic canal can occur.

**Figure 3:**
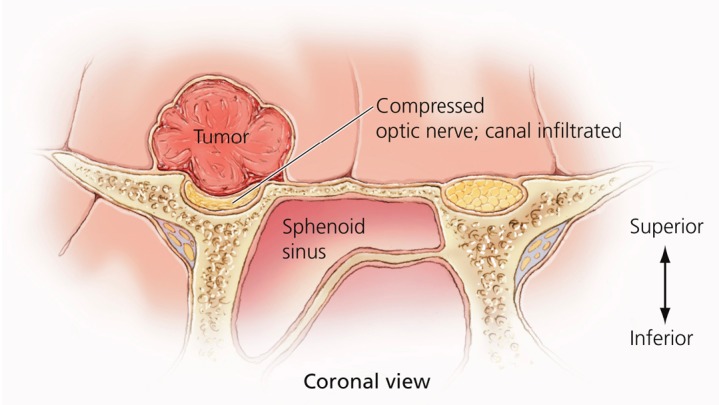
Coronal image of tumor and surrounding structures As tumors enlarge in the cisternal supraclinoid segment, they displace the optic nerve and ICA inferiorly and medially, covering these structures from view to the operating surgeon via an intradural approach.

Meningiomas of the anterior clinoid process are uncommon tumors, acknowledged by most experienced surgeons to be among the most challenging meningiomas to completely remove due to their propensity to encase the internal carotid artery (ICA) and its branches, and invade the cavernous sinus and the optic canal [1-4]. In many cases, the tumor is densely adherent to the carotid artery, rendering complete tumor removal impossible, even in experienced hands [4-7] (Figures 1-3).

To date, most surgical outcome studies of meningioma patients have focused on presenting and analyzing outcomes of a cohort of patients undergoing surgery for a specific type of meningioma as single unified patient cohort [1-6, 8-10]. However, it is hard to argue that any group of skull base meningiomas represent a unified group of uniform pathologic anatomy. While some skull base meningiomas present as a localized mass, others present as a diffuse mass, infiltrating the cavernous sinus, encasing vessels, and invading cranial nerve foramina. Most skull base surgeons are well aware that not all clinoid meningiomas are the same. However, due to the rarity of these lesions, it has been difficult to sub-stratify and sub-analyze these lesions differently based on differing radiographic features. Thus, the literature to date has generally not analyzed outcomes for clinoidal meningiomas in the same way that skull base surgeons think of them when they are planning an operation.

While complete tumor removal, if possible, is the goal of all meningioma surgery, the evolution of stereotactic radiosurgery and 3D-conformal radiotherapy as effective, less invasive treatment options for addressing residual tumor postoperatively has paradoxically made evidence-based intraoperative decision in these cases more complex [11-14]. We would argue that in present neurosurgical practice, surgery of these complex, multi-faceted lesions can best be described as a series of risk-benefit comparisons in which the surgeon weighs the risks and benefits of surgically removing each portion of the tumor against leaving all or part of that portion of the tumor and treating with radiosurgery, or simply observing the residual disease with serial imaging.

In this article, we summarize our institutional experience removing these uncommon and challenging skull base meningiomas. We have specifically targeted our analysis towards radiographically characterizing the frequency of specific radiographic characteristics of surgically treated lesions at our institution. We further characterize the significance of these tumor characteristics for surgical decision-making and clinical outcome, with a specific emphasis on analyzing the impact of the decision to open the optic canal on visual outcomes.

## Materials and methods

### Patient population

The patients were adults (age ≥ 18 years) who underwent surgery at the University of California at San Francisco (UCSF) between 1991 and 2009, had preoperative and postoperative (< 72 hours) magnetic resonance imaging (MRI), and had at least one year of clinical follow-up. Patients with hemangiopericytomas were excluded from the study. Patients were included in this analysis only if radiosurgery did not seem an appropriate alternative treatment option given the clinical and radiographic characteristics of the specific cases. In general, these cases involved tumors greater than 2.5 cm in the largest diameter, tumors with imaging features concerning for higher histologic grade (i.e. irregular borders, an indistinct interface with the cortical surface), tumors growing rapidly on serial imaging, and tumors with significant symptoms referable to mass effect.

This study was approved by the UCSF Committee on Human Research (approval #H7828-29842-03). Informed patient consent was obtained for all patients undergoing treatment.

### Microsurgical technique, surgical strategy, and perioperative management

Intraoperative neuronavigation was used routinely in order to help identify the internal carotid artery (ICA) displayed as a red object using merged 2D magnetic resonance angiography with axial T1-weighted images. This was helpful for the larger tumors where the ICA was enveloped by the tumor. For most cases, a cranio-orbital skull base approach was used while attempting a Simpson Grade 1 resection whenever possible. Preoperative embolization was generally not performed for these tumors given that these are often supplied largely by small ICA perforators, which cannot be sacrificed. Tumors were generally approached using a standard front temporal craniotomy or with a two part frontotemporal orbitozygomatic osteotomy. The decision whether or not to perform extradural anterior clinoidectomy and unroof the optic canal performed was based on attending practice patterns (some attending physicians unroof the optic canal routinely as was the practice of the senior author, others routinely do not).

**Figure 4:**
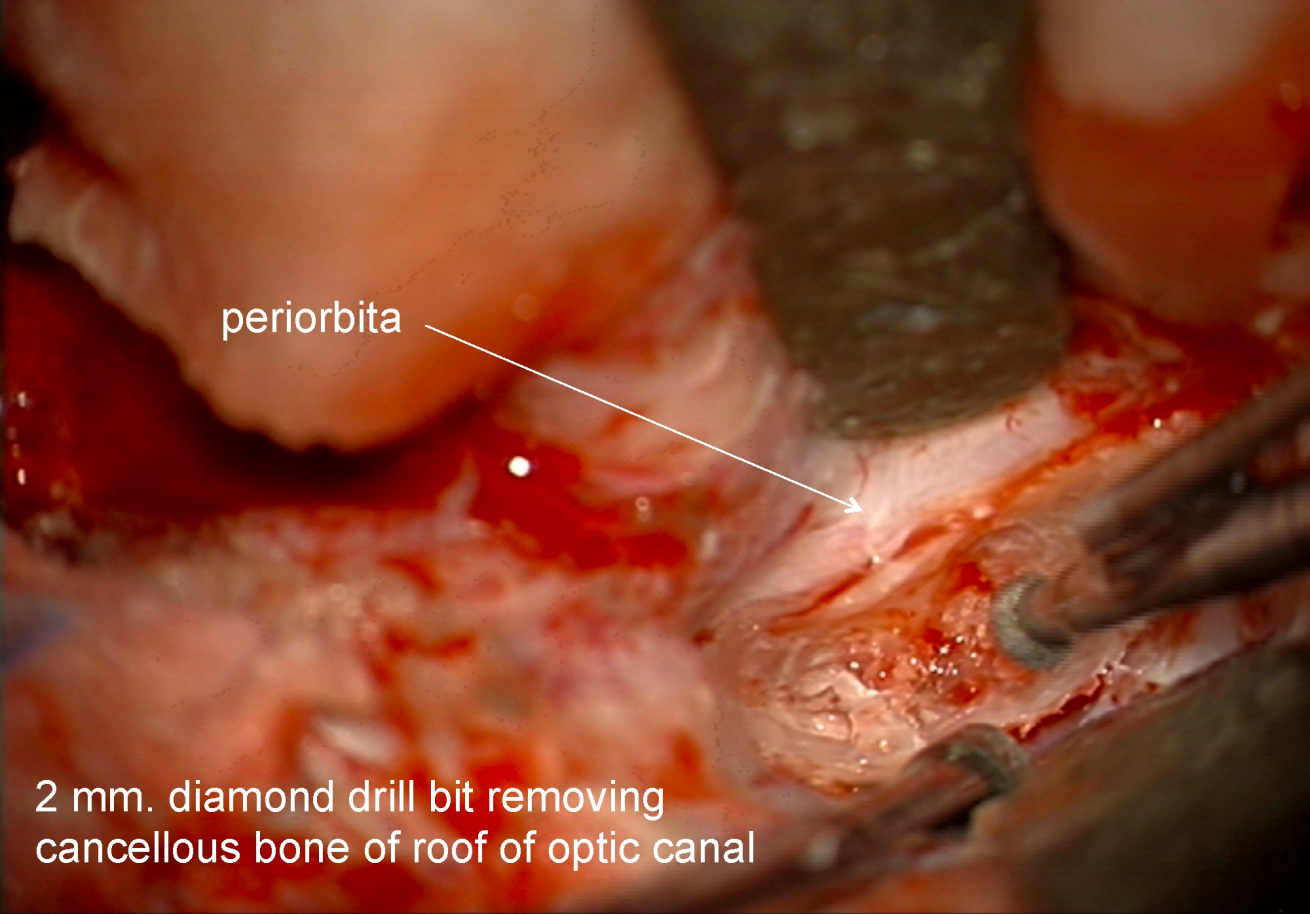
Roof of optic canal Intraoperative image of the initial drilling of the roof of the optic canal.

**Figure 5:**
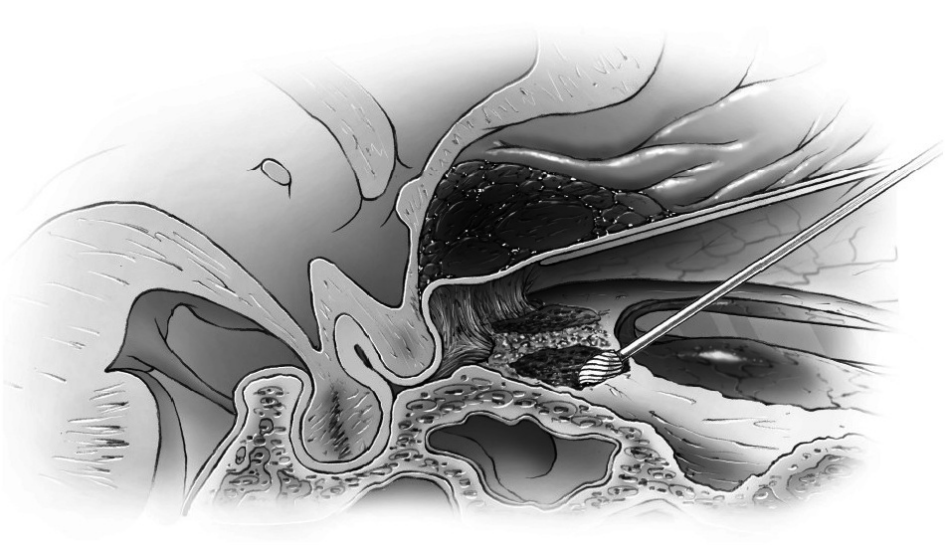
Extradural approach Artists depiction of this phase of the extradural approach.

For those cases in which the optic canal was drilled out, an extradural method was used. If there was involvement of the infra-clinoid region then an extradural clinoidectomy was performed after opening the optic canal. The senior author’s rule was: in order to do a safe clinoidectomy you first need to open the optic canal, but you don't need a clinoidectomy to only open the optic canal. To open the optic canal, the extradural dissection proceeded medially along the sphenoid wing until the orbitomeningeal fold was identified. The first 6-8 mm of the fold were cut to allow exposure of the length of the clinoid. The bone of the roof of the orbit toward the orbital apex was removed using a 2 mm diamond drill bit with constant irrigation (Figures 4-5). 

**Figure 6:**
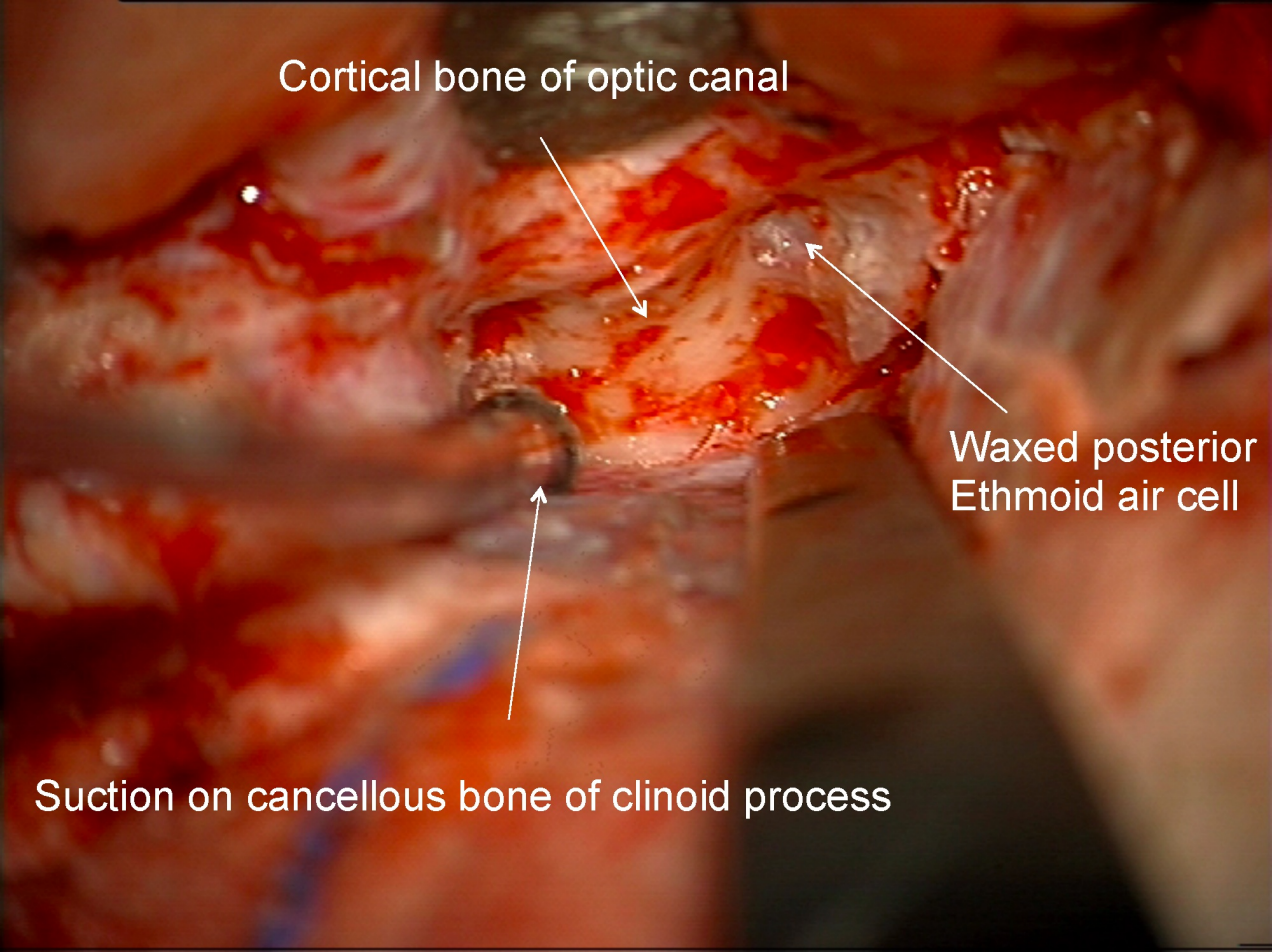
Optic Canal Exposed Intraoperative image of cortical bone of roof of optic canal exposed. Medial border of  waxed ethmoid air cells and lateral border of cancellous bone of clinoid marked with arrows.

**Figure 7:**
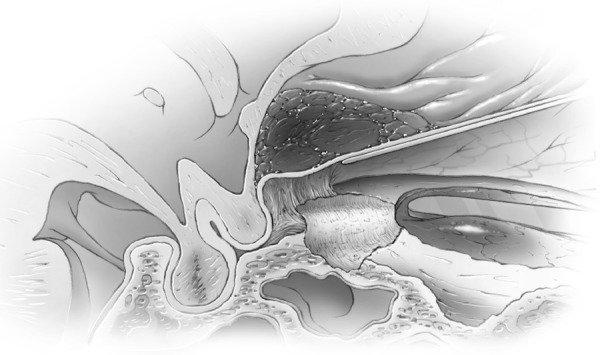
Optic canal exposed Artists illustration of image.

**Figure 8:**
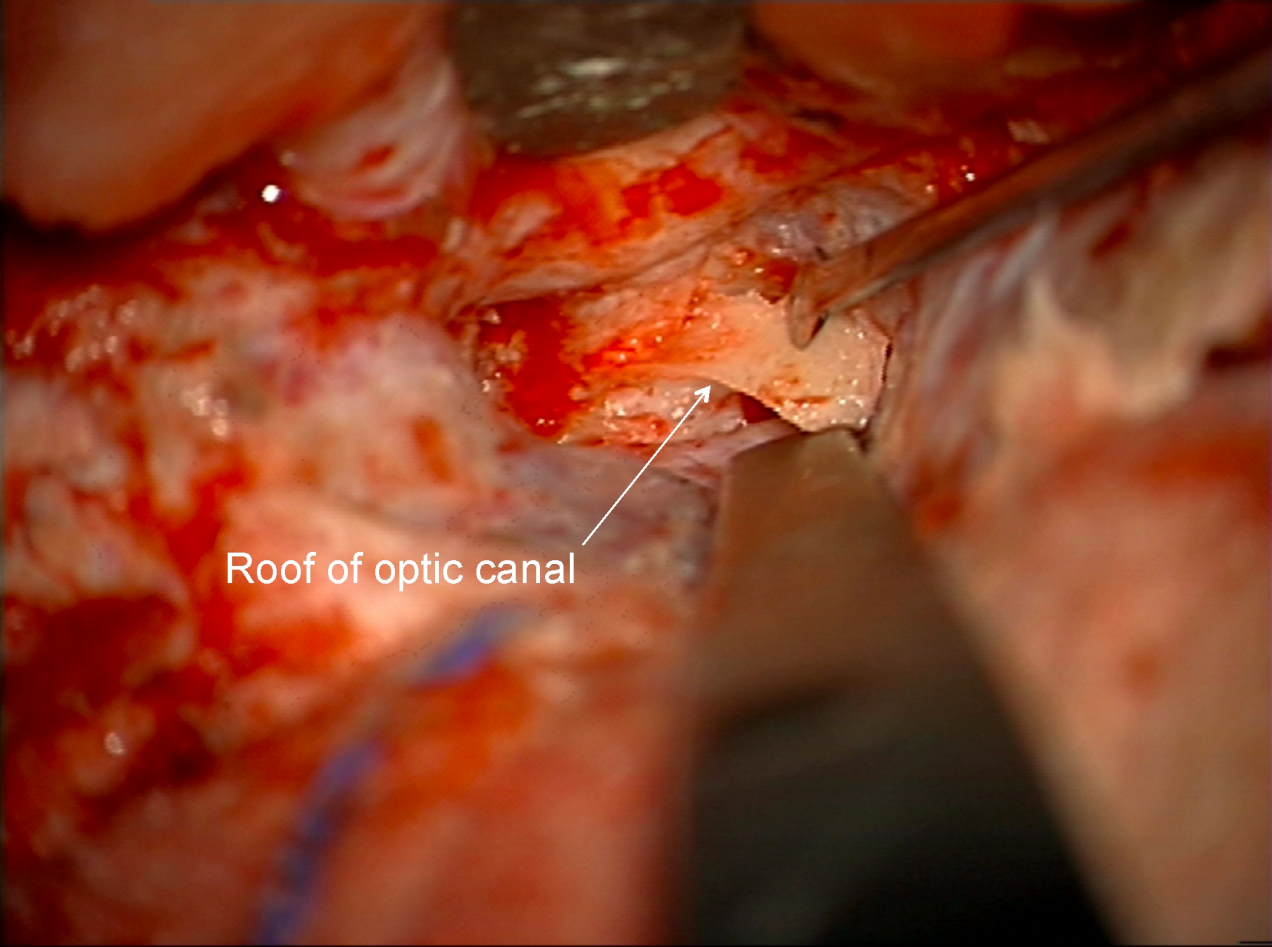
Removal optic canal roof After drilling a medial and lateral gutter through cortical bone of the optic canal, the central 2/3 of the roof is dissected off optic nerve sheath using micro instrument (Rhoton #4).

**Figure 9:**
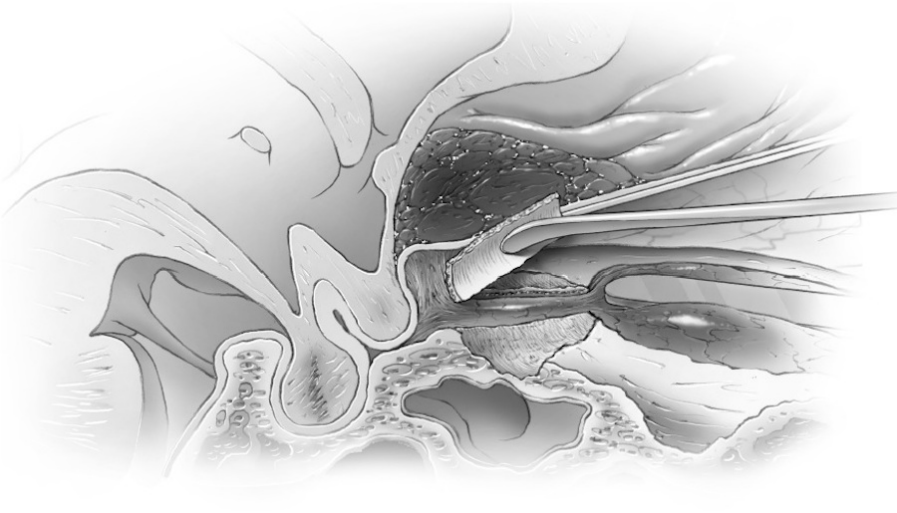
Removal optic canal roof Artist's depiction of image.

Ultrasonic aspirators were never used for bone removal around the canal or clinoid. A trough was created on the medial and lateral side of the canal using the marrow space of the clinoid as the lateral marker for the lateral limit of the canal and posterior ethmoid air cells as the medial limit (Figures 6-7) and then the central two-thirds of the roof was removed with a #4 Rhoton microdissector (Figures 8-9). 

**Figure 10:**
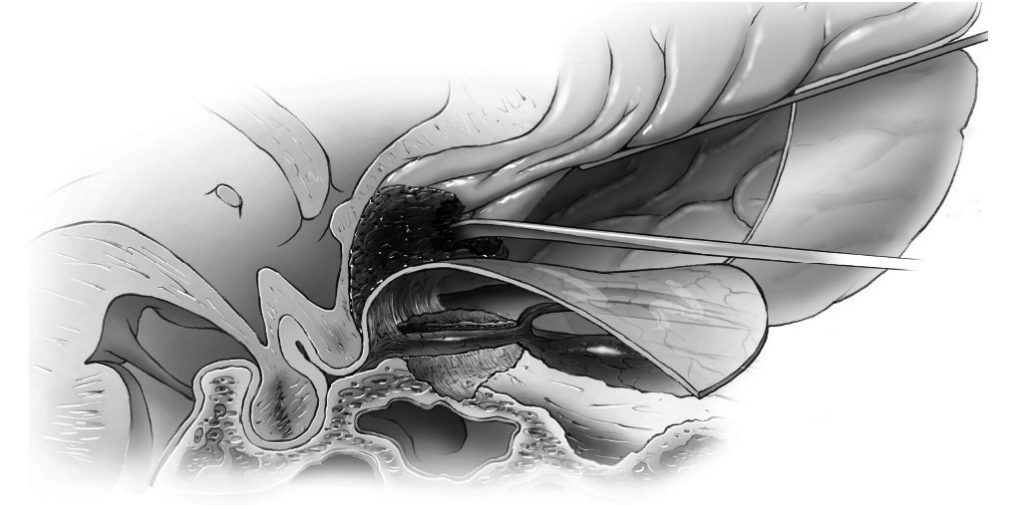
Intradural tumor removal Artist's depiction of first steps in intradural tumor removal towards base near clinoid/canal region.

**Figure 11:**
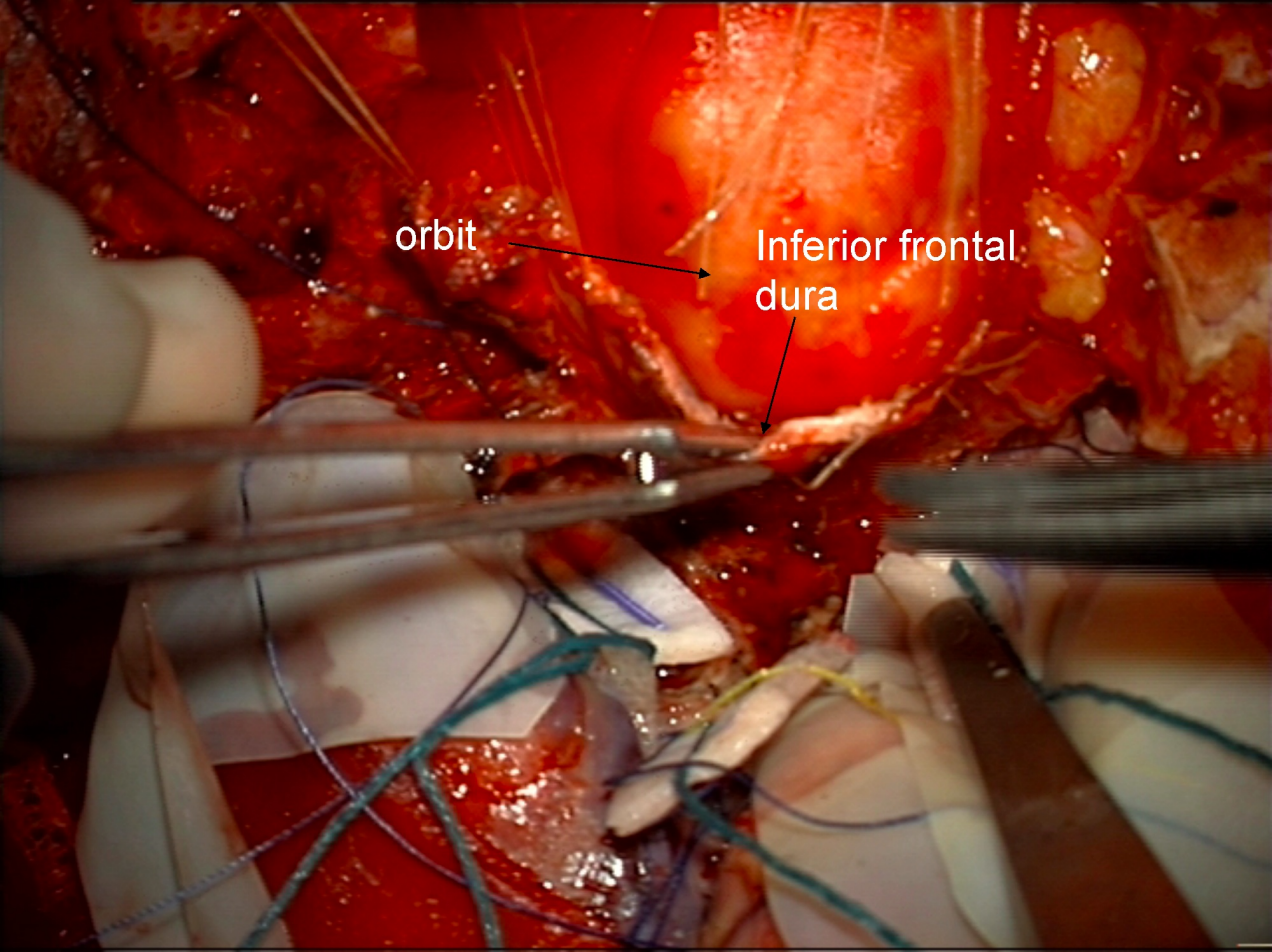
Incision of dura Intraoperative image of initial incision of inferior frontal dura after tumor debulking.

**Figure 12:**
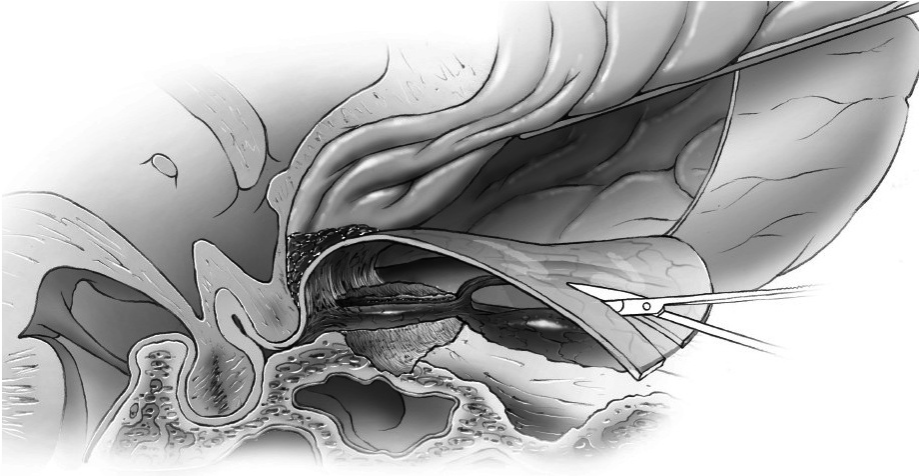
Incision of dura-B&W Artist's depiction of image.

It is thought that limiting drilling of the cortical bone of the roof of the optic canal to the medial and lateral sides may reduce the chance of optic nerve injury from the transmission of mechanical energy or heat. If clinoidectomy was required, it was completed at this stage using the posterior lateral aspect of the optic canal as a marker for the medial drilling of the base of the optic strut. Intradural exposure of the tumor from an inferior frontal approach with tumor debulking until such time as the basal parts of the tumor needed attention (Figure 10). Here, the basal frontal dura was incised back towards the optic canal and nerve sheath (Figures 11-12).

**Figure 13:**
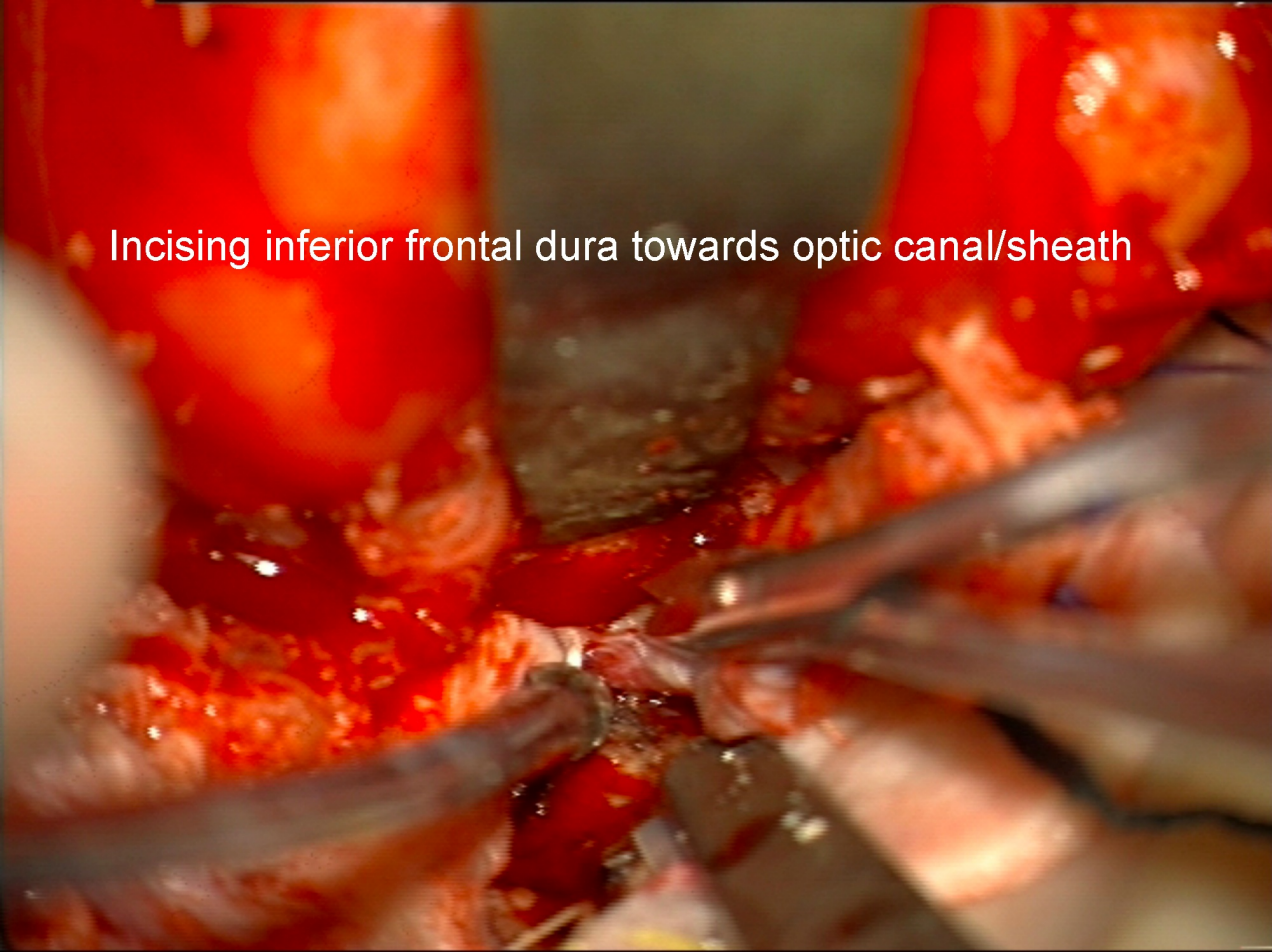
Incising inferior frontal dura near optic canal Image of deeper incision of inferior frontal dura, which becomes continuous with falciform ligament and dura of the optic nerve sheath.

**Figure 14:**
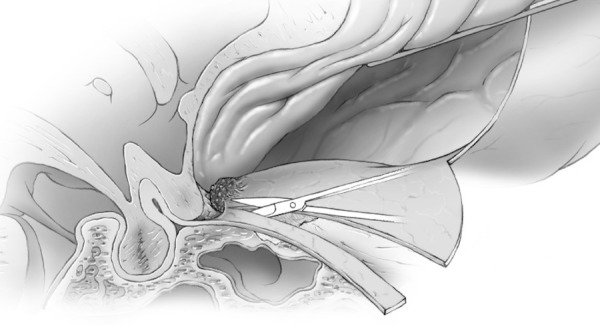
Incising inferior frontal dura near optic canal Artist's depiction of image.

**Figure 15:**
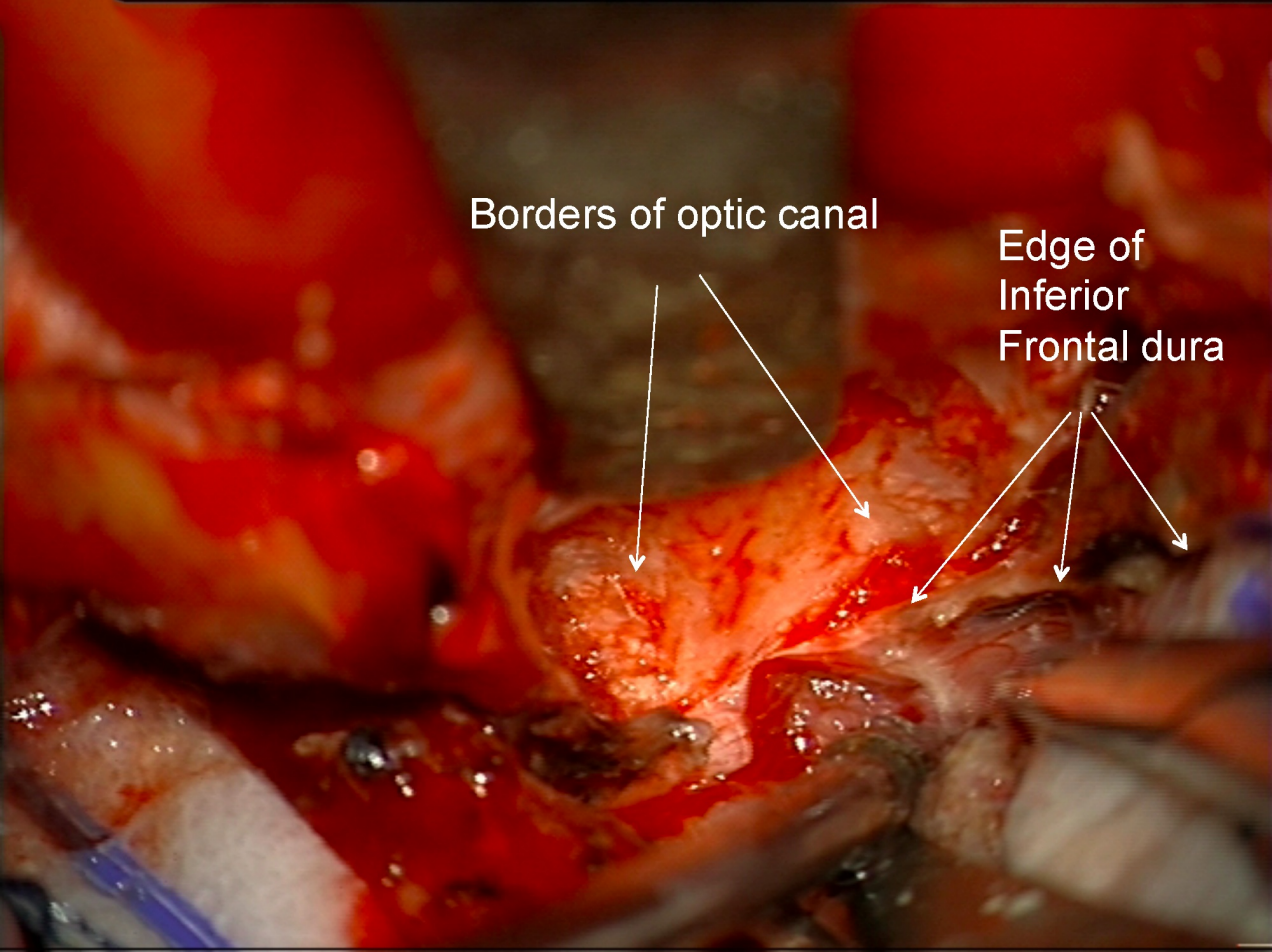
Opening falciform ligament Incision completed through the inferior frontal dura, falciform ligament, and into the proximal optic nerve sheath.

Proximally, the dura becomes continuous with the falciform ligament and the optic nerve sheath in the optic canal (Figures 13-15).

**Figure 16:**
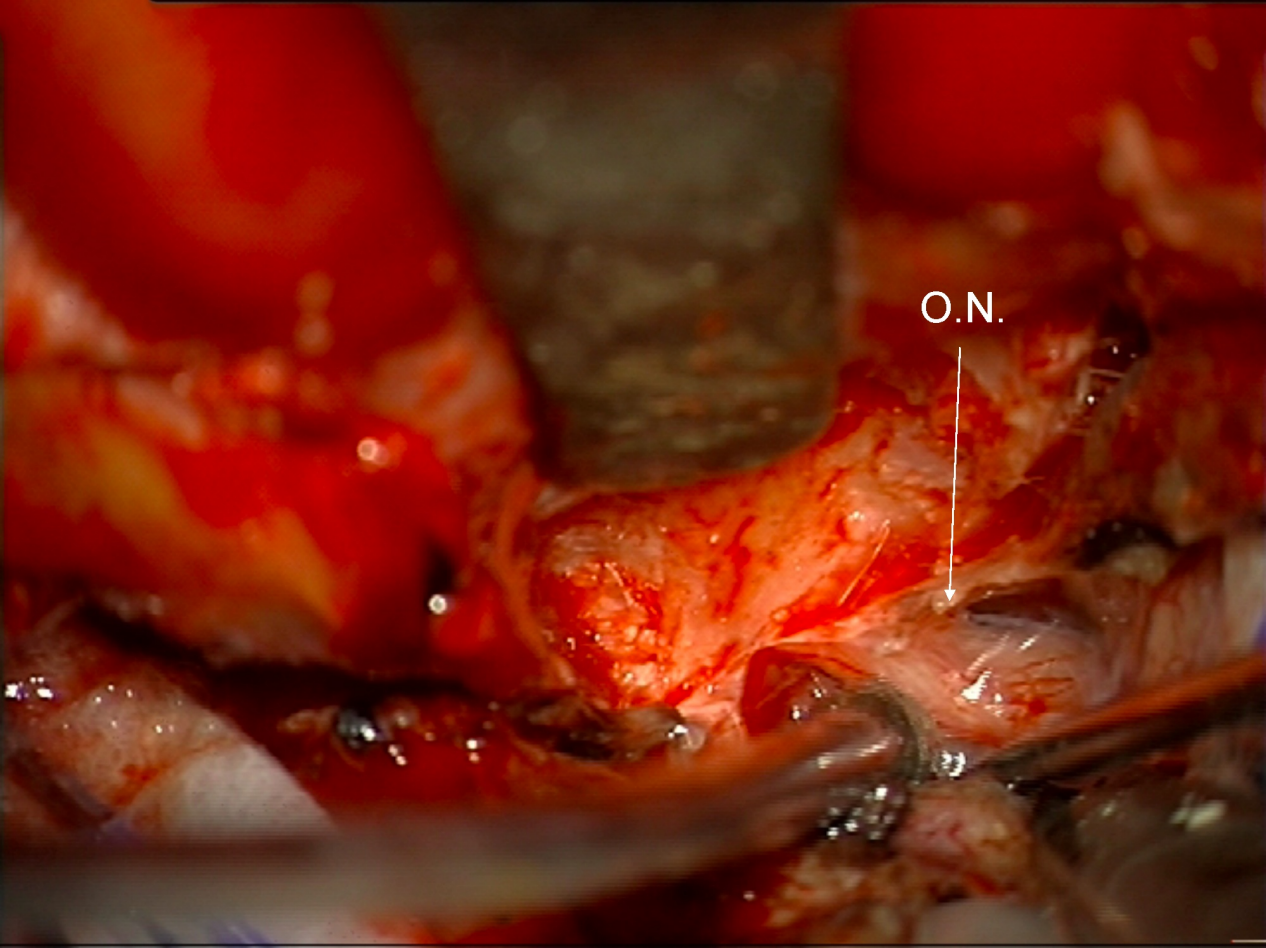
Identification of displaced optic nerve Optic nerve identified, displaced posteriorly, inferiorly and medially by tumor. This would not be seen until the end of the dissection if an intradural dissection were performed without opening of the optic canal.

The optic nerve can be identified pushed down and/or medially (Figure 16).

**Figure 17:**
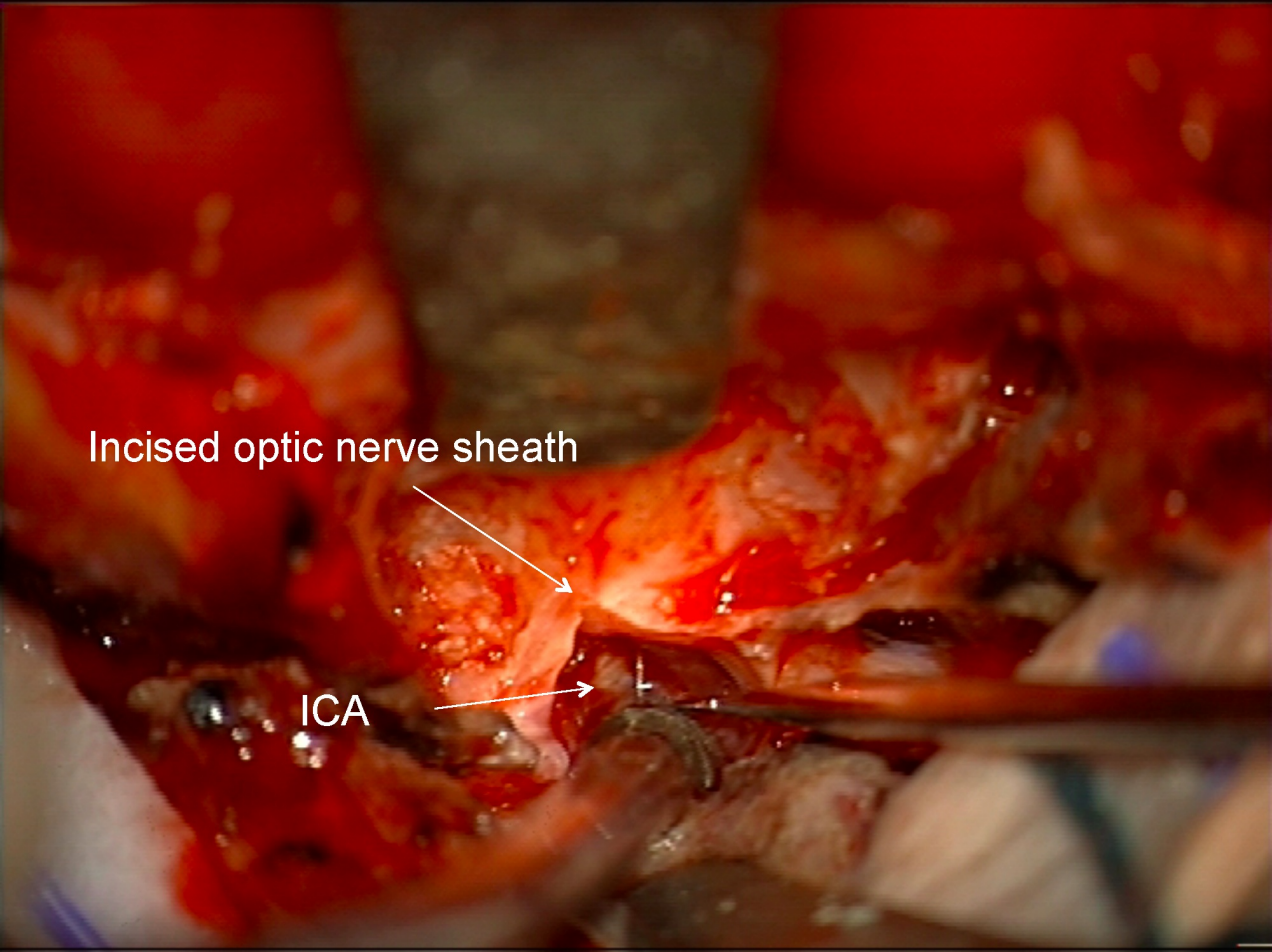
Identification of ICA After identifying the optic nerve, the ICA can be found after removing tumor entering the lateral and superior part of the optic canal.

Just deep to the displaced optic nerve, the ICA can be found after removing the tumor in the proximal, superior, and lateral aspect of the optic canal (Figure 17).

**Figure 18:**
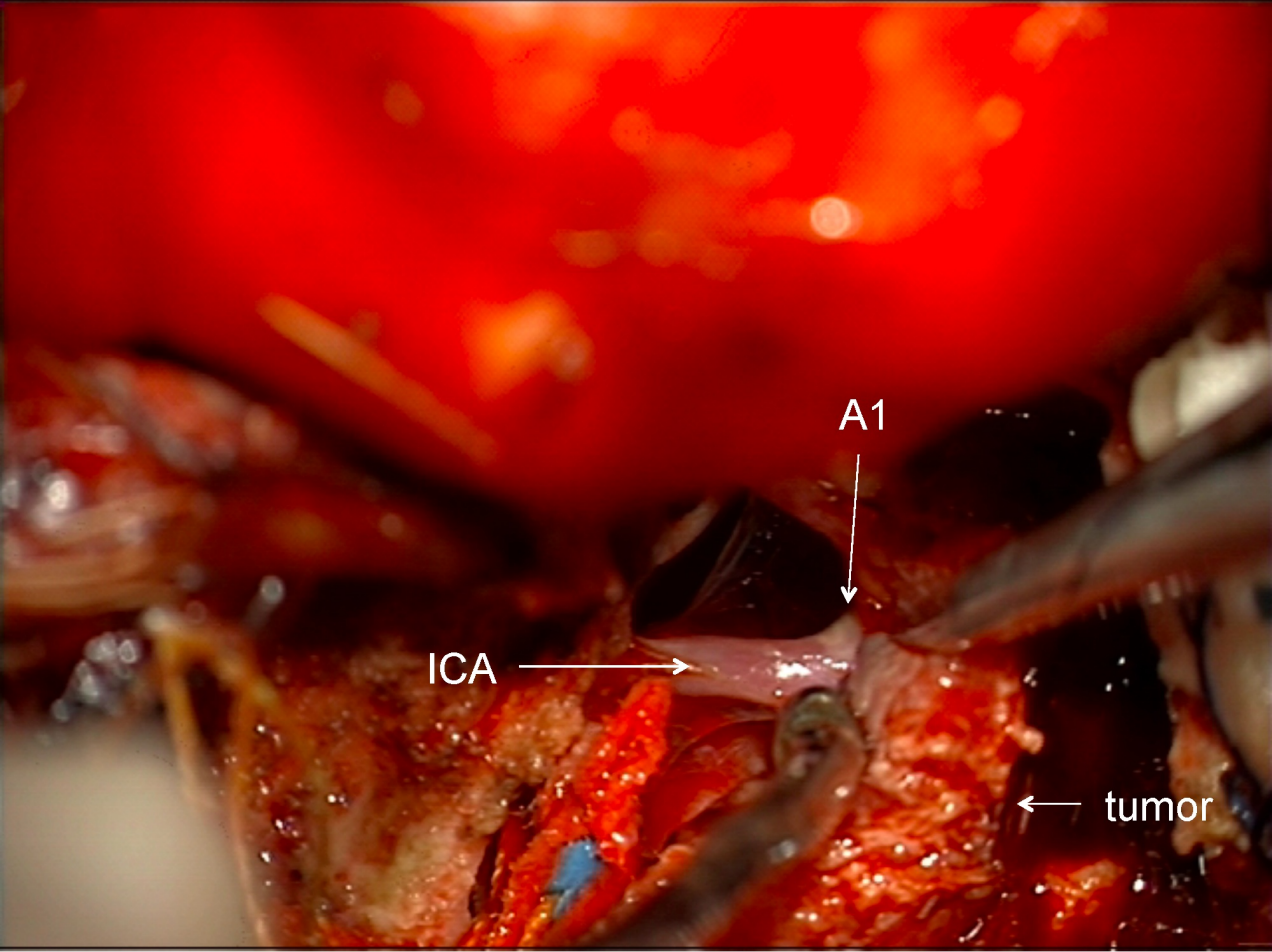
Dissection of ICA Dissection of subarachnoid planes performed along ICA after detachment of tumor from the base and having identified both the optic nerve and ICA early in the dissection.

Now the two critical structures to be preserved have been found and the tumor detached from the base, both assisting with further dissection of the infra-clinoid and cisternal tumor (Figure 18).

Once intradural in general, the tumor was debulked from within using an ultrasonic aspirator. Careful attention was paid to identifying and respecting the arachnoid plane at the tumor-brain interface, which facilitates complete resection and minimizes pial vessel injury. Whenever possible, the involved dura was resected or cauterized.  

While the goal of the operation from the onset was total tumor removal, the discovery of significant tumor adherence to the cranial nerves or the internal carotid artery, or significant invasion of the cavernous sinus, generally prompts us to seek near total resection, leaving a small amount of tumor in the involved region.

Intraoperatively, all patients received decadron (10 mg), mannitol (1 g/kg), and ceftriaxone (1 or 2 gm) at the time of incision. Postoperatively, all patients were cared for in a neuro-intensive care unit for one day before returning to the ward. On postoperative day 2, a prophylactic dose of enoxaparin (40 mg SC each day) was initiated in all patients and continued for one week. Routine use of venous thrombosis prophylaxis was not started until after 2001 [15]. The incidence of postoperative intracranial hemorrhage was no different in the patient groups before or after prophylaxis was begun [16]. Irrespective of preoperative seizure history, all patients were also loaded with an antiepileptic agent at the time of surgery (Dilantin initially, Keppra more recently), which was continued for one week postoperatively and then discontinued.

### Data collection

Preoperative MR imaging was reviewed for each patient in order to confirm the diagnosis of a meningioma arising from the anterior clinoid process, to determine the tumor dimensions, to determine whether the tumor was based on the superior or inferior clinoid surface, and to determine whether vascular encasement, invasion of the cavernous sinus or optic canal, or sellar involvement were present.

We routinely perform formal assessments of visual function using formal visual acuity testing and formal perimeter field testing, both pre- and postoperatively. Improvement in visual function was defined as > 30% reduction in visual field deficit and/or meaningful improvement in visual acuity on postoperative examination. Worsening in visual function was defined as any new visual field cut, or any significant decline in visual acuity postoperatively. Visual function was defined as “unchanged” if no change or minor change occurred between tests. All patients were seen by ophthalmologists for visual follow-up, and the visual outcome was obtained from written objective assessments of visual improvement and patient reports. Conversion from written or printed forms to electronic medical records did not allow for scanning of visual fields into the EMR in all but one case. Paper records were subsequently destroyed after the digital conversion, limiting our ability to display postoperative visual field patterns.

Central pathology review was performed on the basis of the most recent World Health Organization (WHO) guidelines [17]. Clinical data were collected from the patient records and telephone interviews. All clinical assessments were performed by a neurosurgeon. In each case, the extent of resection and Simpson Grade [18-19] were determined using a combination of the surgeon’s assessment and MR imaging. 

### Statistical analysis

Binary variables were compared using Pearson’s χ^2^ test. Continuous variables were compared using an independent samples t-test or ANOVA, after statistical confirmation of normality. Continuous variables are presented as mean ± SE. Statistical tests were considered significant when p < 0.05 after correcting for multiple comparisons using the Bonferroni method. 

## Results

### Patient and tumor demographics

We identified 29 patients with anterior clinoid meningiomas who underwent surgical resection at our institution between 1991 and 2007. The demographic characteristics of individual patients are listed in Table 1. The median length of follow-up was 7.5 years (range: 2.0 to 16.9 years). The median patient age was 53 years old at the time of surgery (range: 21 to 78 years old). The patient population was largely female, which is not unusual for a series of meningioma patients. Twenty-seven of 29 patients had WHO Grade 1 meningiomas. All patients underwent surgery as opposed to radiotherapy either due to large tumor size, proximity of the tumor to the optic apparatus, or both.

**Table 1:** Patient Demographics Individual patient demographics for the patients in this study

#	Age	Gender	Size (cm)	WHO grade	OZ?	Simpson Grade	Embo?	Optic Canal Drilled?	Upfront Radiosurgery?	F/u (yr)	Recur?
1	21	M	4.4 x 4.0 x 4.9	1	No	4	No	No	No	10.6	No
2	22	F	3.0 x 3.0 x 3.5	1	No	4	No	No	No	12.9	No
3	30	M	4.0 x 3.8 x 3.6	1	Yes	2	No	Yes	No	5.4	No
4	33	F	1.0 x 1.5 x 1.0	1	No	2	No	No	No	8.0	No
5	35	F	4.5 x 4.1 x 3.6	1	Yes	4	No	No	No	9.5	No
6	62	F	2.7 x 1.9 x 3.0	1	Yes	4	Yes	Yes	Yes	7.4	No
7	40	F	1.4 x 1.3 x 2.0	1	Yes	4	No	No	No	6.3	No
8	42	F	2.8 x 2.9 x 3.2	1	Yes	4	No	Yes	No	7.9	No
9	44	F	3.2 x 2.6 x 2.6	1	Yes	1	No	Yes	No	6.8	No
10	46	M	2.5 x 2.2 x 1.7	1	Yes	4	No	Yes	No	7.6	No
11	50	F	2.0 x 2.0 x 2.0	1	No	1	No	No	No	12.8	Yes
12	51	F	2.0 x 2.3 x 1.5	1	Yes	4	No	Yes	No	7.7	No
13	52	M	2.1 x 2.2 x 1.5	1	Yes	4	No	No	No	4.9	No
14	53	F	3.3 x 2.4 x 1.6	1	Yes	4	No	Yes	No	9.3	No
15	53	F	3.8 x 3.0 x 3.2	1	Yes	4	No	No	No	5.1	No
16	55	F	3.0 x 3.5 x 4.2	1	No	4	No	Yes	No	16.9	No
17	56	F	2.0 x 1.5 x 2.0	1	Yes	4	No	Yes	Yes	6.2	No
18	56	M	3.4 x 4.2 x 3.5	1	Yes	4	No	No	No	9.7	Yes
19	56	F	2.2 x 1.1 x 1.5	1	Yes	4	No	Yes	No	6.4	No
20	59	F	5.7 x 4.7 x 4.2	1	Yes	1	No	Yes	No	6.1	No
21	62	F	1.5 x 1.5 x 1.5	1	Yes	4	No	No	No	6.3	No
22	63	F	4.9 x 4.8 x 4.3	1	Yes	4	No	Yes	No	5.9	No
23	63	F	2.5 x 2.5 x 2.5	2	Yes	4	No	Yes	No	2.0	No
24	64	F	4.4 x 3.8 x 3.1	1	Yes	4	No	Yes	No	4.3	No
25	67	F	2.0 x 2.0 x 1.5	2	Yes	3	No	Yes	No	9.7	No
26	34	F	3.4 x 3.0 x 1.9	1	Yes	4	No	Yes	No	6.5	No
27	72	F	5.5 x 5.0 x 4.5	1	Yes	4	No	Yes	No	9.9	No
28	77	F	2.0 x 1.5 x 2.2	1	No	2	No	No	No	8.7	No
29	78	F	3.6 x 2.8 x 3.0	1	Yes	4	No	Yes	No	7.5	No

### Relationship between preoperative radiographic characteristics and presenting symptoms

The relationship between presenting symptoms and radiographic tumor characteristics as summarized in Table 2. We found that 25/29 of tumors had a supraclinoidal origin, while 4/29 were infraclinoidal in origin. All four patients with an infraclinoidal origin demonstrated cavernous sinus invasion while only 3/25 patients with supraclinoidal tumor origin did. About half of tumors (15/29) invaded the optic canal, and slightly less than half of these tumors encased the supraclinoid carotid artery (14/29). Sellar invasion was present in 12/29 of the patients.

**Table 2:** Imaging Characteristics and Symptoms The frequency of various imaging characteristics of anterior clinoid meningiomas, and the relationship between these findings and various presenting symptoms.

Table 2		MS	HA	Tremor	Hearing	Ptosis	Vision	None
	#	3	5	1	2	1	20	3
Optic Canal Invasion	Absent	2	2	0	1	0	8	2
	Present	1	3	1	1	1	12	1
Cavernous Sinus	Absent	2	5	0	1	1	15	3
	Present	1	0	1	1	0	5	0
Origin	Supraclinoid	3	5	1	1	1	19	1
	Infraclinoid	0	0	0	1	0	1	2
Vessel Encasement	Absent	1	3	0	0	1	11	2
	Present	2	2	1	2	0	9	1
Sellar Invasion	Absent	1	4	0	1	1	11	2
	Present	2	1	1	1	0	9	1

Interestingly, while patients with optic canal invasion by tumor usually presented with decreased preoperative vision (12/15), over half of the patients without optic canal invasion did as well (8/14), suggesting that radiographic optic canal invasion is not necessary for visual compromise in these tumors. Also interesting was the complete absence of hypopituitarism in these patients despite sellar invasion being present in a large number of patients. Cranial nerves, III, IV, and/or VI, were only present in one patient preoperatively, which is similarly interesting given the proximity of these tumors to the nerves of the superior orbital fissure.

### Relationship between preoperative radiographic characteristics and extent of resection

Similar to others [2], we found gross total resection was seldom safely achievable in these patients. Simpson Grade 1 resection was achieved in three patients, Grade 2 resection was achieved in three patients, and Grade 3 resection was achieved in one patient. We were unable to achieve gross total resection in any cases with an infraclinoidal tumor origin or cavernous sinus invasion (Table 3). We were, however, able to obtain gross total resections (Simpson Grades 1-3) in a few patients with optic canal invasion (3/15), vessel encasement (2/14), and sellar invasion (2/10).

**Table 3:** Imaging & Extent or Resection The relationship between imaging characteristics and extent of resection achieved in these patients.

		Simpson Grade				
		1	2	3	4	Total
	#	3	3	1	22	29
Optic Canal Invasion	Absent	2	2	0	10	14
	Present	1	1	1	12	15
Cavernous Sinus	Absent	3	3	1	15	22
	Present	0	0	0	7	7
Origin	Supraclinoid	3	3	1	18	25
	Infraclinoid	0	0	0	4	4
Vessel Encasement	Absent	2	2	1	10	15
	Present	1	1	0	12	14
Sellar Invasion	Absent	2	2	1	12	17
	Present	1	1	0	10	12

Despite the frequent need for subtotal resection for these tumors, we found that these tumors seldom progressed following subtotal resection, even without radiosurgery. Of the 22 patients in this series who received a Simpson Grade IV resection, two patients underwent radiosurgery for the residual disease in the cavernous sinus shortly following surgery. Of the remaining 20 patients not undergoing upfront adjuvant postoperative radiosurgery, only 1/20 (5%) has experienced documented growth of their residual tumor during the follow-up period (Table 1). This cohort interestingly includes two WHO Grade 2 tumors, which have not recurred to date.

### Postoperative visual outcome with or without optic canal unroofing

The optic canal was unroofed in 18/29 of patients in this series. In 11/29 of patients, the meningioma was removed, and when possible, the dura overlying the anterior clinoid process was coagulated; however, the optic canal was not unroofed, and tumor invasion into the optic canal was not addressed surgically. Notably, all five patients experiencing visual improvement underwent optic canal unroofing, while three of four patients experiencing visual worsening did not (Table 4). While statistical significance is difficult to achieve in cohorts of this size, these data did display a statistical trend towards improved outcomes with unroofing the optic canal (χ^2^ p=0.13).

**Table 4:** Optic Canal Opening and Vision Visual outcomes of patients who underwent optic canal unroofing and those who did not

Optic Canal Unroofed?	Improved	Same	Worse	Total
Yes	5	12	1	18
No	0	8	3	11

To address the possibility that extent of resection might impact visual outcomes, we compared visual outcomes in patients stratified by the Simpson Grade of resection (Table 5). Again, it is difficult to draw firm conclusions in a cohort this size; however, these data do not obviously suggest a significant relationship between subtotal resection and improved/worsened visual outcomes, as some patients receiving Simpson Grade 4 resection experienced improved visual outcomes, while others experienced worse outcomes.

**Table 5:** Simpson Grade and Visual Outcome A summary of extent of resection and visual outcome in the 29 patients in this study.

Simpson Grade	Improved	Same	Worse	Total
1	0	3	0	3
2	1	2	0	3
3	0	1	0	1
4	4	14	4	22

### Surgical morbidity and mortality after resection of anterior clinoid meningiomas

Seven of 29 patients (24%) in this series experience at least one medical, neurosurgical, or neurologic complication resulting from the surgical procedure. Four of these seven patients experienced worsening visual function as described in the previous section. One of the remaining patients suffered a retraction injury causing word finding difficulties, which largely had resolved at long-term follow-up. One patient developed a venous infarction, which caused facial nerve weakness that also resolved by six months postoperatively. One patient developed new hydrocephalus, which eventually required ventriculoperitoneal shunting. There were no wound complications and no medical complications (i.e. DVT/PE’s, UTI’s, cardiac, renal, pulmonary, hepatic, etc.) in this cohort. The six-month mortality rate in these patients was 0%.

In addition, one patient (patient #17) (Table 1) who had a tumor with significant cavernous sinus involvement and proximity to the optic nerve, underwent craniotomy and removal of the components of the tumor near the optic apparatus and in the optic canal. The craniotomy was uneventful. Four months postoperatively, she subsequently underwent Gamma Knife radiosurgery with 15 Gy to the 50% isodose line administered to the cavernous sinus disease. Eight months post-Gamma Knife, she presented with intermittent motor symptoms and eventually underwent angiography demonstrating complete occlusion of the cavernous carotid. Given that she had good collateral filling through the posterior communicating artery, she was treated with aspirin alone with good symptom resolution.

## Discussion

In our opinion, anterior clinoid meningiomas should be conceptually thought of as being really three different tumors in close proximity: the cisternal portion, the cavernous/carotid portion, and the optic canal portion, although not all of these portions are present in all cases. In this conceptual framework, each of these three “tumors” poses different issues regarding their proximity to the optic apparatus, their relationship to important neurovascular structures, and the challenges of the surgical maneuvers necessary to remove tumor from the relevant anatomic region. Thus, the decision to remove the tumor from the optic canal represents a risk-benefit decision comparing the relative merits of drilling out the optic canal and removing the tumor, versus leaving tumor behind and observing it. With this question formally posed, the relative merits of different treatment approaches can potentially be systematically studied, and the decision about whether this surgical technique is a worthwhile risk can be made based on data targeted at specifically answering this question. Due to the rarity of these lesions, it is difficult for any one center to definitively answer this question alone, and thus, our study represents the first formal contribution of data towards this answer.

The present study presents data regarding the frequency of various preoperative anatomic characteristics and clinical outcomes for a moderately sized series of patients treated surgically for anterior clinoid meningiomas at our institution. While our series adds to a growing literature regarding outcomes of patients undergoing surgery for these difficult lesions [1-6, 8-10, 20], due to the rarity of these lesions, it is unlikely that any one center treating these lesions can acquire enough experience with anterior clinoid meningiomas to definitively answer important questions, such as “Should surgeons drill out the optic canal and remove tumor from the canal?”, and “What is the fate of residual tumor left in the cavernous sinus or on the ICA?” Due to the variability of anatomic presentation of skull base meningiomas, the rarity of many meningioma subtypes (in this case, clinoid meningiomas), and the long time period that these patients need to be observed in the postoperative period, it is very likely that these questions can only be answered through the collaborative efforts of multiple centers over many years. It is important, however, that such data collection follows a standardized, detailed, and rational methodology, so that important confounding variables can be controlled for, and that well-conducted studies to address these important questions can be structured. 

Our analysis of surgical outcomes of patients with anterior clinoid meningiomas aimed to provide data for two major questions regarding these tumors. The first question was whether it is wise to unroof the optic canal and attempt to remove the tumor in these cases. Interestingly, we found that all patients who experienced visual improvement underwent optic canal unroofing while most patients whose vision worsening postoperatively did not. These data are certainly not definitive; however, they suggest that optic canal roofing is at least not clearly a bad idea and might be helpful. Interestingly, two of the patients whose vision improved did not have obvious radiographic evidence of tumor invasion into the canal, and the optic canal unroofing was performed as part of the extradural clinoidectomy, suggesting that merely the fact that tumor removal from the optic canal decompressed the optic nerve cannot explain the visual improvement in these cases. Possible benefits of optic canal unroofing in these cases include protection of the optic nerve from vibratory and thermal injury during surgery, elimination of a point of kinking of a compressed optic nerve, and reduction of pressure around the optic nerve in the perioperative period when tumor and/or nerve swelling might occur [1-3, 6]. The latter might be of particular importance given the frequent need to leave behind tumor in these cases. It is important to note that these findings may not necessarily extend to optic canal invasion from other meningiomas, such as those arising from the tuberculum sella, as the different origin of these tumors might cause different tumor-optic nerve orientations and different arachnoidal planes than those seen with anterior clinoid meningiomas [1-2]. As such, these data deserve independent analysis.

An additional question we sought to study in our dataset was: Given the frequent need for subtotal resection in these cases, is upfront postoperative adjuvant radiosurgery or radiotherapy indicated to prevent growth of the residual disease [14]? We found that even without conformal radiotherapy or radiosurgery, most residual tumors did not regrow over a period of several years of follow-up, suggesting that adjuvant therapy can usually be avoided with close imaging follow-up. Given the close proximity of these tumors (and their post-surgical remnants) to the highly radiosensitive optic nerve [11, 14], the fact that radiation can often be avoided or delayed in many patients with subtotal WHO Grade I tumors is not a trivial point. This concept is further highlighted by the post-radiosurgery carotid occlusion experienced by patient #17, which highlights the fact that radiosurgery, while generally safe and effective, is not entirely benign when administered in this region and should be reserved for patients with residual disease demonstrating growth on follow-up imaging studies. On the other hand, observation is also not entirely free of risk, and we recommend performing annual follow imaging on these patients, as a delay in the re-treatment of persistent tumor regrowth can leave the patient with a much more difficult to manage problem.

## Conclusions

In conclusion, we present data from our series of patients with surgically treated anterior clinoid meningiomas, which suggests that while a conservative approach to these lesions can still provide reasonable rates of tumor control; even without upfront radiosurgery/radiotherapy to the residual disease, some aggressive maneuvers, such as unroofing the optic canal, might be beneficial. At a minimum, these data contribute to what we hope will be the beginning of a collaborative effort towards a systematic approach to assessing the risks and benefits of techniques we utilize in skull base surgery.
